# Molecular Mechanism Underlying the Action of Substituted Pro-Gly Dipeptide Noopept

**Published:** 2016

**Authors:** Y. V. Vakhitova, S. V. Sadovnikov, S. S. Borisevich, R. U. Ostrovskaya, T. A.Gudasheva, S. B. Seredenin

**Affiliations:** State Zakusov Institute of Pharmacology , Baltiyskaya Str., 8, 125315, Moscow, Russia; Institute of Biochemistry and Genetics of Ufa Scientific Centre RAS, Prospekt Oktyabrya, 71, 450065 , Ufa, Russia; Ufa Institute of Chemistry RAS, Prospekt Oktyabrya, 71, 450065, Ufa, Russia

**Keywords:** Noopept, transcriptional factors, HIF-1, hypoxia, HIF-prolyl hydroxylase 2, docking, neuroprotection

## Abstract

This study was performed in order to reveal the effect of Noopept (ethyl ester of N-phenylacetyl-*L*prolylglycine, GVS-111) on the DNA-binding activity of transcriptional factors (TF) in HEK293 cells transiently transfected with luciferase reporter constructs containing sequences for CREB, NFAT, NF-κB, p53, STAT1, GAS, VDR, HSF1, and HIF-1. Noopept (10 μM) was shown to increase the DNA-binding activity of HIF-1 only, while lacking the ability to affect that of CREB, NFAT, NF-κB, p53, STAT1, GAS, VDR, and HSF1. Noopept provoked an additional increase in the DNA-binding activity of HIF-1 when applied in conditions of CoCl2-induced HIF- 1 stabilization. The degree of this HIF-positive effect of Noopept was shown to be concentration-dependent. Piracetam (1 mM) failed to affect significantly any of the TF under study. The results of molecular docking showed that Noopept (*L*-isomer), as well as its metabolite, *L*-isomer of N-phenyl-acetylprolyl, unlike its pharmacologically ineffective *D*-isomer, is able to bind to the active site of prolyl hydroxylase 2. Taking into account the important role of the genes activated by HIF-1 in the formation of an adaptive response to hypoxia, data on the ability of Noopept to provoke a selective increase in the DNA-binding activity of HIF-1 explain the wide spectrum of neurochemical and pharmacological effects of Noopept revealed before. The obtained data allow one to propose the HIF-positive effect as the primary mechanism of the activity of this Pro-Gly-containing dipeptide.

## INTRODUCTION

Noopept (ethyl ester of N-phenylacetyl-*L*-prolylglycine) was designed as a drug at State Zakusov Institute of Pharmacology. The synthesis of the drug is based on the original hypothesis of peptide design, according to which structures similar to known psychotropic agents are reproduced using appropriate amino acids [1]. The non-peptide prototype of Noopept is the nootropic drug Piracetam. The pharmacological activity of the new compound turned out to be generally similar to the activity of Piracetam; but, it manifestes itself at doses 1,000 times lower than those for Piracetam [2, 3]. Moreover, Noopept has more pronounced anxiolytic [4] and neuroprotective properties [5-7].

A clinical study of Noopept (registration number 015770) confirmed the nootropic effects established experimentally. In patients with mild cognitive impairment of cerebrovascular and post-traumatic origin, the drug decreased cognitive impairment, showed an anxiolytic effect, and vegetostabilizing activity (www. noopept.ru).

The mechanism of Noopept action has been studied since its synthesis. It has been established that the drug increases the expression of NGF and BDNF in the hippocampus [8], exhibits choline-positive properties at behavioral and neuronal levels [9], reduces oxidative stress and enhances the activity of antioxidant systems [7, 10], and represses kinases pSAPK/JNK and pERK1 induced by stress [11]. However, the study of the primary interactions of Noopept with more than 100 known receptors conducted according to our protocol by the CEREP company (France) did not lead to the expected identification of the primary targets. At the same time, the wide range of the neurochemical and pharmacological effects of Noopept prompted the further search for its targets.

In order to obtain more exhaustive information on the targets of Noopept, we analyzed *in vitro *the influence of the drug on the DNA-binding activity of pharmacologically significant biological targets, the transcription factors (TF) CREB, NFAT, NF-**κ**B, p53, STAT1, GAS, VDR, HSF1, and HIF-1. Having identified the selective influence of Noopept on HIF-1, we examined the effect of the drug on the activity of this transcription factor under conditions mimicking the hypoxia *in vitro*.

## EXPERIMENTAL

**Cell culturing**

A HEK 293 cell line (human embryonic kidney cells; Russian Collection of Cell Cultures, Institute of Cytology, RAS, St. Petersburg) was used in the study. The cells were cultured at 37°C, 5% CO_2_ in DMEM medium (Biolot, Russia) with 10% fetal bovine serum (Sigma, USA), 2 mM *L*-glutamine, 50 μg/ml gentamycin sulfate, and 2.5 μg/ml amphotericin B (PanEco, Russia).

The influence of Noopept on the DNA-binding activity of transcription factors was examined using luciferase reporter constructs containing binding sites for CREB, NFAT, NF-κB, p53, STAT1, GAS, VDR, HSF1, and HIF-1 according to [12].

For transfection, HEK293 cells were seeded (4×10^3^ cells/per well) in 96-well plates in 100 μl of a DMEM medium containing 10% fetal bovine serum and 2 mM *L*-glutamine without an antibiotic. Reporter vector constructs containing binding sites for the transcription factors CREB, NFAT, NF-κB, p53, STAT1, GAS, VDR, HSF1, and HIF-1 were obtained on the basis of the pTL-Luc plasmid vector (Panomics, USA; carries the *Photinus pyralis *luciferase gene) [13]. The HEK293 cells were transiently transfected with the constructs using the Lipofectamine 2000 reagent (Invitrogen, USA) according to the manufacturer’s recommendations. The medium was replaced with a medium containing an antibiotic (DMEM, 10% fetal bovine serum, 2 mM *L*-glutamine, 50 μg/ml gentamicin sulfate) 6 hours after transfection, and the studied drugs (Noopept 10 μM; Piracetam, 1 mM) were added after 18 h. The cells were incubated in the presence of either Noopept or Piracetam for another 24 hours. Luciferase activity in cell lysates was determined using a Dual Luciferase Reporter Assay System (Promega, USA) on the plate reader 2300 EnSpire® Multimode Plate Reader (Perkin Elmer, USA). Co-transfection with plasmid pRL-TK (Promega, USA) encoding the *Renilla reniformis* luciferase gene was used as an internal control for transfection. The values of the *P. pyralis *luminescence were normalized to the luminescence of *R. reniformis* in each measurement.

Experimental simulation of hypoxia was performed using CoCl_2_, as pharmacological inducer of hypoxia, causing stabilization of HIF-1 [14]. The luciferase construct for the analysis of HIF-1 activity contains four copies of a consensus sequence 5’-ACGTG- 3’, an HIF-1 protein-binding site (HIF-1-Luc construct). The cells transfected with the plasmid vector HIF-1-Luc were preincubated with Noopept for 8 hours (final concentrations 1, 10, and 100 μM; double administration every 4 hours), then the hypoxia inductor CoCl_2_ was added at a working concentration of 50 μM, and combined incubation with Noopept and CoCl_2_ proceeded for an additional 16 hours. After that, luciferase activity was determined as described above.

**Statistical analysis**

The arithmetic mean of the values obtained for two repeats in each experiment in a series of three independent experiments and the standard error of the mean value were calculated using the Statistica 6.1 software (StatSoft Inc., USA). Experimental groups were compared using a paired Student’s t-test for dependent samples.

**Molecular docking**

The three-dimensional structure of the target protein prolyl hydroxylase 2 (PHD2; hypoxia-inducible factor-*L*-proline, 2-oxoglutarate: oxygen oxidoreductase, [1.14.11.29]; PDB code: 2G19) in a complex with the native inhibitor (ZINC code: 24800213; IC_50_ 1.4 μM) was used for construction and validation of the docking model [15, 16]. Noopept and its *D*-enantiomer (ZINC codes: 1542824 and 3812682, respectively), Noopept metabolite *L*-N-phenylacetyl-proline (ZINC code: 76075), and stereoisomers of the previously described [31] prolyl hydroxylase inhibitor (PA2L and PA2D) (*Table*) were considered as ligands. The geometrical parameters of the majority of the molecules were extracted from the ZINC database [17] or modeled using the ChemCraft v1.7 software [18] and optimized by the HF/6-311G(d,p) method on the GAUSSIAN 09 C.01 software [19]. Preparation of target and ligand structures for docking, as well as docking, was performed using the LeadIT 2.1.8 software [20]. All quantum chemical calculations were performed on a cluster supercomputer at the Ufa Institute of Chemistry, RAS.

**Table 1:** *In silico *estimation of the energies of interaction between ligand and receptor

Ligand code	Ligand structure	ΔG_FlexX_, kJ/mol^1^	RMSD^2^	ΔG_HYDE_, kJ/mol^3^	LE^4^
ZINC24800213	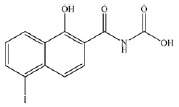	-31.8	0.42	-63	0.79 (H)
ZINC1542824_L	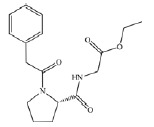	-17.0	0.48	-44	0.45 (HA)
ZINC3812682_D	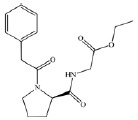	-17.5	1.10	-42	0.42 (HA)
ZINC76075_L	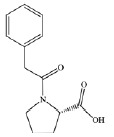	-24.1	0.47	-38	0.53 (H)
PA2_L	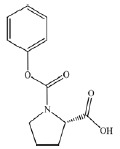	-27.2	0.55	-49	0.55 (H)
PA2_D	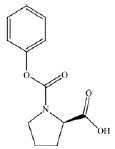	-28.2	0.73	-41	0.47 (HA)

^1^ΔG_FlexX_ – free binding energy, kJ/mol.

^2^RMSD – root-mean square deviation of ligand position in active site.

^3^ΔG_HYDE_ – affinity energy between ligand and binding site, kJ/mol.

^4^LE – ligand efficiency (LE = |ΔG_HYDE_|/*N *[22, 23], where *N *– number of heavy, i.e. not hydrogen atoms), where ligand efficiency can be evaluated as H – high efficiency, HA – higher than average efficiency [22].

**Fig. 1:**
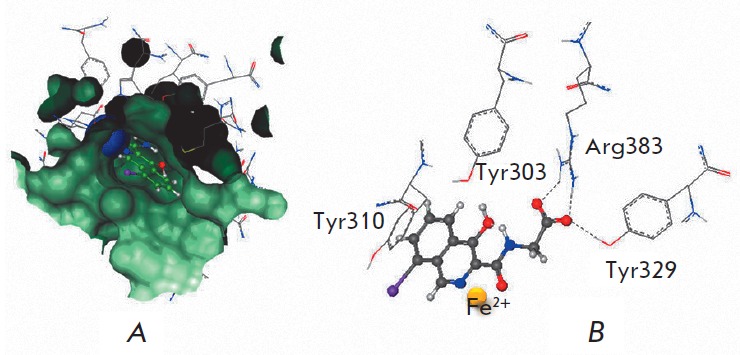
Analysis of the active site of prolyl hydroxylase enzyme 2. *A *– active center occupied by “native” ligand.* B *– interaction between inhibitor and amino acid residues of the enzyme (docking solution)

The surrounding area of the native inhibitor (ZINC: 24800213) with adjacent amino acid residues is 6.5 A and contains Arg383, Tyr310, Tyr303 and Fe^2+^. Analysis of the 2G19 enzyme active center showed that Arg383 and Tyr329 form hydrogen bonds with the carboxyl group of the ZINC 24800213 native inhibitor; Tyr310 and Tyr303 form a π–π electron interaction with the aromatic rings of the ligand. The amino acid residues Trp389, Trp258, Met299, and Ile256 form a hydrophobic pocket (*Fig. 1*). All water molecules were removed from the active center during preparation of the enzyme structure for the docking procedure. Re-docking of the native ligand into the PHD2 enzyme active site accurately reproduces the mode of binding between the ligand and enzyme that has been determined crystallographically. The root-mean-square deviation is 0.44 A. The subprogram FlexX [21] allows to perform the procedure of ligand docking (*Table*) and estimate the energy of binding between the ligand and receptor in the active site. The number of docking decisions can be large enough, and the choice of an optimal solution is based on the minimum value of the binding energy in combination with a minimum root-mean-square deviation (RMSD) value when the ligand is in the binding site. Next, the selected position is subjected to further calculation: assessment of the affinity energy between the ligand and binding site (ΔG_HYDE_, kJ/mol) and evaluation of ligand effectiveness [22, 23]. A detailed algorithm of the calculation is described in [22]. It is noted that it is optimal to use two successive stages of the selection of the leader compound among the ligands.

## RESULTS AND DISCUSSION

**Fig. 2:**
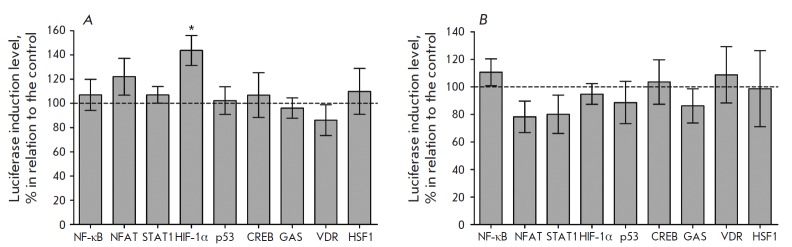
Effect of Noopept, 10 μM (*A*), and piracetam, 1 mM (*B*), on the basal DNA-binding activity of the transcriptional factors NF-κB, NFAT, STAT1, HIF-1, p53, CREB, GAS, VDR, and HSF1 *in vitro*. The statistical significance of the differences was assessed using a paired Student’s t-test for dependent samples (*n *= 3, *p  < 0.05)

**Fig. 3:**
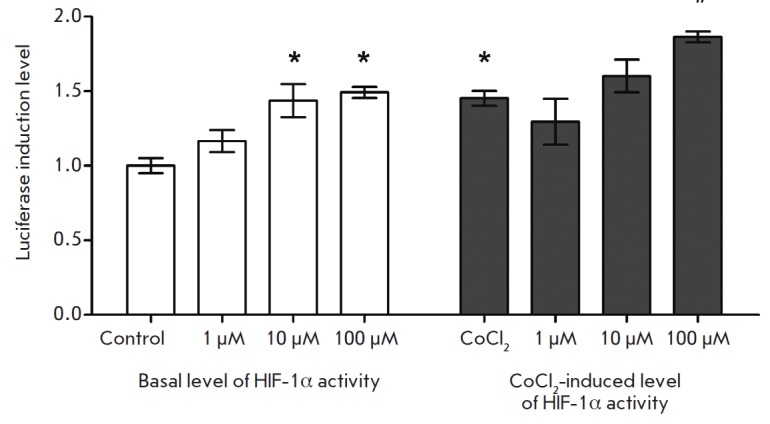
The effect of Noopept on the basal and induced activity of HIF-1. “Control” group – values of the basal activity of HIF-1 in unstimulated cells; “CoCl_2_” group – HIF-1 activity values in CoCl_2_-stimulated cells. The data are presented as arithmetic mean ± standard error of the mean (*n *= 3; *p  < 0.05 with respect to “control” group; *#p * < 0.05 with respect to “CoCl_2_” group)

The data presented in *Fig. 2A* indicate that incubation with Noopept at a concentration of 10 μM for 24 hours enhances the DNA-binding activity of HIF-1 by 43% and does not caused any statistically significant changes in the DNA-binding activity of the factors CREB, NFAT, NF-κB, p53, STAT1, GAS, VDR, and HSF1. As follows from the data presented in *Fig. 3*, Noopept at concentrations of 10 and 100 μM increases the level of luciferase induction. It was shown that Piracetam at either an equimolar (10 μM, data not shown) or higher concentration (1 μM) does not cause statistically significant changes in the DNA-binding activity of the studied transcription factors (*Fig. 2B*).

The next stage of the study included the analysis of the influence of Noopept on the DNA-binding activity of HIF-1 in the presence of a pharmacological mimetic of hypoxia CoCl_2_. In full accordance with the well known data conserning CoCl_2_ action, an increase in HIF-1 activity was observed. Addition of Noopept at concentrations of 10 and 100 μM resulted in a further increase in HIF-1-dependent luciferase activity (*Fig. 3*). Thus, it has been established for the first time that Noopept is able to increase both the basal activity of HIF-1 and the activity induced by a pharmacological mimetic of hypoxia *in vitro*.

**Fig. 4:**
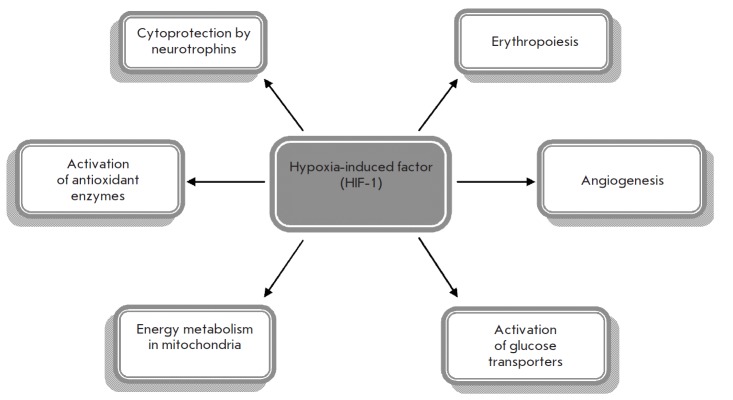
Hypoxia-induced factor HIF-1 and its targets. Modified according to [24]

The factor induced by hypoxia (HIF-1) is a heterodimer composed of two subunits: a HIF-1α subunit sensitive to oxygen and constitutively expressed HIF-1β. Hypoxia promotes an increase in the HIF-1α level, its dimeriation with HIF-1β, mobilization of coactivators (p300/CBP), and binding of this complex to HRE (hypoxia-response element) in the regulatory regions of target genes. In normoxic conditions, oxygen-dependent hydroxylation of proline residues in the HIF- 1α molecule by prolyl hydroxylases is necessary for binding by the component of ubiquitin-protein ligase E3, von Hippel-Lindau (VHL) protein. Ubiquitinated HIF-1α becomes a target for degradation by 26S proteasomes. The asparagine residue at the C-terminal transactivation domain (C-TAD) of HIF-1α is hydroxylated by asparagin hydroxylase (FIH1, factor inhibiting HIF-1) in the presence of oxygen, thereby blocking its interaction with the transcriptional coactivator p300/CBP. Thus, PHD and FIH inactivate HIF-1α in normoxia, suppressing HIF-1-dependent expression of the target genes. PHD and FIH activity decreases under conditions of hypoxia, leading to a decrease in HIF-1α degradation and transcriptional activation of its dependent genes [24]. It has been shown [25] that HIF-1 activates a total of up to 100 genes. *Figure 4* presents the main targets of HIF-1, which include the genes involved in angiogenesis through activation of the vascular endothelial growth factor, enhanced synthesis of erythropoietin, activation of the systems of glucose transport through membranes, cytoprotection by neurotrophic factors, normalization of cell cycle and metabolism at the mitochondrial level, as well as the activity of antioxidant enzymes: superoxide dismutase and catalase. The combination of these effects allows the implementation of an adaptive response to hypoxic exposure. Alongside with this, HIF-1 affects the state of many neurotransmitter systems: it activates the protein responsible for control of GABA receptors (GABARBP) [26] and increases tyrosine hydroxylase activity [27]. The close relation between HIF-1 and cholinergic receptors has been described [28].

As shown in our study, Noopept causes a concentration- dependent increase in the basal DNA-binding activity of HIF-1. Upon stabilization of HIF-1 with CoCl_2_, a chemical inducer of this transcription factor [29, 30], Noopept provides an additional increase in the HIF-1 DNA-binding activity. The effect on HIF-1 is specific for Noopept: the classical nootropic drug Piracetam does not affect the activity of this transcription factor. Noopept enhances the DNA-binding activity of HIF-1 alone, while the activity of other transcription factors (CREB, NFAT, NF-κB, p53, STAT1, GAS, VDR and HSF1) is not increased. Since prolyl hydroxylase is directly involved in HIF-1 deactivation, and proline analogs are described as effective inhibitors of this enzyme [31], it can be assumed that the increase in the DNAbinding activity of HIF-1 by Noopept is associated with the inhibition of this enzyme.

The comparison of the structures of prolyl hydroxylase inhibitors presented by Ma *et al*. [31] with that of Noopept and its metabolites suggests a similarity between the PA2 (benzyloxycarbonyl-Pro) compound and an N-terminal fragment of the Noopept molecule, N-phenylacetyl-Pro (*Table*) [32, 33]. It should be noted that the range of concentrations at which PA2 inhibits prolyl hydroxylase (*K*_i_ = 2.38 μM, EC_50_ = 3.17 μM) [31] is close to the level of effective concentrations for Noopept identified in the present study.

**Fig. 5:**
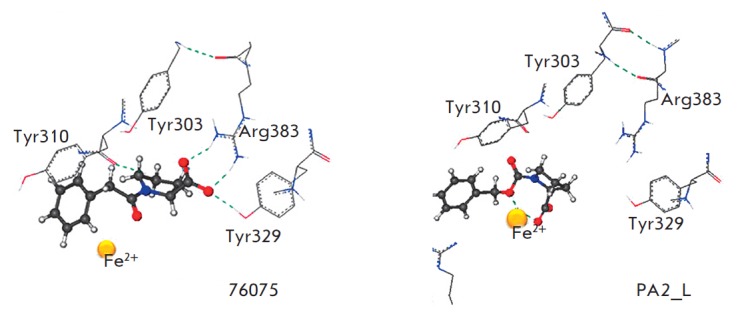
Results of molecular docking: location of ligands in the active site of prolyl hydroxylase (hydrogen bonds are shown in dashed line)

According to the results of the molecular docking, Noopept and *L*-isomer of N-phenylacetyl-Pro binds to the active site of prolyl hydroxylase at a level of efficiency comparable with that of RA2L *L*-isomer (*Table*). The qualitative effectiveness of the ligand is estimated as high. *L*- stereoisomer of N-phenylacetyl-Pro forms hydrogen bonds with Arg383 and Tyr329 in the active site of the enzyme, and the PA2L molecule is coordinated by oxygens around the Fe atom (*Fig. 5*). It should be underlined that the pharmacologically inactive *D*isomer of Noopept has a lower binding energy. Thus, it can be assumed that Noopept and its metabolite, *L*isomer of N- phenylacetyl-Pro, may bind to the active site of prolyl hydroxylase 2 and, probably, inhibit its enzymatic activity. Apparently, the final conclusion requires further experimental study of the effect of Noopept and its metabolite on the activity of prolyl hydroxylase. Possible interaction of Noopept with asparagine hydroxylase also requires additional studies.

Returning to the question of the interaction between Noopept and HIF-1 while lacking that for Piracetam, it should be noted that Noopept is designed as a dipeptide analogue of Piracetam. However, the effective doses of Noopept are three times lower than that of Piracetam [2]. The new drug and its non-peptide prototype demonstrate different spectrum of pharmacological activity. Thus, Noopept facilitated all phases of information processing, while Piracetam influenced mainly initial phases [1]. Noopept exhibited pronounced neuroprotective properties, whereas in Piracetam they, depending on the estimated parameter, were mild [7, 34] or absent [35]. From a pharmacological position, such differences must be based on the specificity of the mechanism of action, which includes the effect of Noopept interaction with HIF-1 identified by us.

Regardless of the details of this interaction, it is important that this Pro-containing dipeptide enhances HIF-1 activity. It is known that activation of the HIF system is now regarded as one of the main mechanisms of neuroprotection during hypoxia, cerebral ischemia, and neurodegenerative diseases [24, 36]. During many years of study, these states have been defined as pharmacological targets for Noopept action on a broad spectrum of relevant experimental models.

Following the ability of Noopept to increase animal survival in hyperbaric hypoxia [37] detected at the beginning of the study of this dipeptide, it has been shown that it reduces the volume of ischemic brain damage in circulatory hypoxia models: for example, cortical photochemically induced thrombosis [6] and ligation of the middle artery [5]. The ability of Noopept to attenuate the severity of oxidative stress was established in neuronal cultures of various types: granular cerebellar cells [35], cortical neuron culture of aborted fetuses with diagnosed Down syndrome [7], PC12 culture [38], SH-SY5Y culture [39], and in vivo experiments on brain tissue and rat plasma [40]. The ability to enhance superoxide dismutase and catalase activity was shown both in the experiment [10] and in clinical conditions [34].

The ability of Noopept not only to eliminate the manifestations of cognitive deficit [41], but to exert also a neuroprotective effect was shown in models of Alzheimer’s disease: it attenuated the disturbance of oxidative processes and calcium homeostasis, enhanced neurogenesis, prevented the tau protein aggregation caused by a fragment of β-amyloid_25-35_ [ 38], and eliminated NGF and BDNF deficit caused by diabetogenic toxin streptozotocin administration into brain ventricles [42]. Noopept is capable of reducing the cytotoxic effect of aggregated α-synuclein in a cell model of Parkinson’s disease [39]. All these numerous effects can be explained by the activation of the HIF-1 transcription factor.

In the past years, we have shown that Noopept has an antidiabetic effect on the streptozotocin model of diabetes [43]. This fact was interpreted by us as a result of the multifactorial metabolic action of the drug: attenuation of the deficit in the antioxidant systems and neurotrophic factors and increased production of pro-inflammatory cytokines typical of diabetes [44]. The results obtained in the present study have drawn our attention to the data on the role of HIF-1 in the development of pathological processes in diabetes mellitus. For instance, the ability of insulin to disrupt HIF-1 formation and the role of this factor deficiency in the development of diabetes type 1 and 2 and its complications have been reported [45]. The impaired function of the glucose transport system GLUT1 and GLUT3 through cellular barriers observed in HIF-1 deficiency promotes the development of insulin resistance in both Alzheimer’s disease and diabetes mellitus [46]. The involvement of HIF-1 in the expression of incretins, important factors of pancreatic β-cell cytoprotection, has been proved [47]. A summary of these data allows to suggest that the HIF-1-positive effect of Noopept participates in the realization of its anti-diabetic effect, including the newly identified one by us ability to increase the level of incretin, a glucagon-like peptide-1 (GLP-1) [44].

## CONCLUSIONS

The data obtained on the ability of the effective nootropic and neuroprotective drug Noopept to cause an increase in the DNA-binding activity of HIF-1 allow one to advance a novel interpretation of the wide spectrum of its action: namely, assume that the HIF-1-positive effect of the drug can be considered as the primary mechanism of its action. Clarification of the molecular mechanisms underlying the HIF-1-positive action of Noopept certainly requires further investigation; but the presence of this effect definitely has a significant value, since it allows one to explain almost all known to date effects of Noopept and, probably, the effects of other biologically active Pro-Gly peptides. These data provide additional evidence for current concepts of the importance of the components of the HIF-1-dependent signaling pathway and the compensation processes activated by this transcription factor in the mechanisms of neuroprotection.

## Abbreviations

CREB: cAMP responsive element-binding protein
NFAT: nuclear factor of activated T-cells
NF-κB: nuclear factor kappa-light-chain-enhancer of activated B-cells
STAT1: signal transducer and activator of transcription
GAS: interferon-γ-activated sequence
VDR: vitamin D3 receptor
HSF1: heat shock transcriptional factor 1
HIF-1: hypoxia-inducible factor 1

## References

[1] Gudasheva T.A. (2011). Bulletin of the Russian Academy of Medical Sciences..

[2] Seredenin S.B., Voronina T.A., Gudasheva T.A., Ostrovskaya R.U., Rozantsev G.G., Skoldinov A.P., Trofimov S.S., Halikas J., Garibova T.L. (1995). Biologically active N-acylprolyldipeptides having antiamnestic, antihypoxic effects. Patent 5.439.930 USA..

[3] Gudasheva T.A., Voronina T.A., Ostrovskaya R.U., Rozantsev G.G., Vasilevich N.I., Trofimov S.S., Kravchenko E.V., Skoldinov A.P., Seredenin S.B. (1996). Eur. J. Med. Chem..

[4] Ostrovskaya R.U., Seredenin S.B., Voronina T.A., Molodavkin G.M., Gudasheva T.A. (2006). In Animal models in biological psychiatry Ed. Kalueff A.V. N.Y.: Nova Sci. Publ. Inc.,.

[5] Gavrilova S.A., Us K.S., Ostrovskaya R.U., Koshelev V.B. (2006). Eksp. Klin. Farmakol..

[6] Ostrovskaya R.U., Romanova G.A., Barskov I.V., Shanina E.V., Gudasheva T.A., Victorov I.V., Voronina T.A., Seredenin S.B. (1999). Behav. Pharmacol..

[7] Pealsman A., Hoyo-Vadillo C., Seredenin S.B., Gudasheva T.A., Ostrovskaya R.U., Busciglio J. (2003). Int. J. Dev. Neurosci..

[8] Ostrovskaya R.U., Gudasheva T.A., Tsaplina A.P., Vakhitova Yu.V., Salimgareeva M.H., Yamidanov R.S., Seredenin S.B. (2008). Bull. Exp. Biol. Med...

[9] Ostrovskaya R.U., Mirzoev T.H., Firova F.A., Trofimov S.S., Gudasheva T.A., Grechenko T.N., Gutyrchik E.F., Barkova E.B. (2001). Eksp. Klin. Farmakol..

[10] Mendzheritskiy A.M., Lysenko A.V., Demyanenko S.V., Prokofyev V.N., Gudasheva T.A., Ostrovskaya R.U. (2003). J. Neurochemical..

[11] Ostrovskaya R.U., Vakhitova Y.V., Salimgareeva M.H., Yamidanov R.S., Sadovnikov S.V., Kapitsa I.G., Seredenin S.B. (2010). Eksp. Klin. Farmakol..

[12] Phippard D., Manning A.M. (2003). Methods Mol. Biol..

[13] Salimgareeva M.H., Sadovnikov S.V., Farafontova E.I., Zaynullina L.F., Vakhitov V.A., Vakhitova Y.V. (2014). Prikladnaya biokhimiya i microbiologiya..

[14] Piret J.P., Mottet D., Raes M., Michiels C. (2002). Ann. N.Y. Acad. Sci..

[15] Rose P.W., Prlić A., Bi C., Bluhm W.F., Christie C.H., Dutta S., Green R.K., Goodsell D.S., Westbrook J.D., Woo J. (2015). Nucleic Acids Research.

[16] McDonough M.A., Li V., Flashman E., Chowdhury R., Mohr C., Liénard B.M., Zondlo J., Oldham N.J., Clifton I.J., Lewis J. (2006). Proc. Natl. Acad. Sci. USA..

[17] Irwin J.J., Shoichet B.K. (2005). J. Chem. Inform. Model..

[18] Zhurko G.A., Zhurko D.A. (2013). ChemCraft..

[19] Frisch M.J., Trucks G.W., Schlegel H.B., Scuseria G.E., Robb M.A., Cheeseman J.R., Scalmani G., Barone V., Mennucci B., Petersson G.A. (2010). Gaussian 09, Revision C.01, Gaussian Inc., Wallingford CT, 2010..

[20] Claussen H., Dramburg I., Gastreich M., Hindle S., Kaemper A., Kramer B., Lilienthal M., Mueller G., Rarey M., Wefing S. (2014). LeadIT. V. 2.1.8 BioSolveIT CmbH, 2014.

[21] Rarey M., Kramer B., Lengauer T., Klebe G. (1996). J. Mol. Biol..

[22] Schneider N., Hindle S., Lange G., Klein R., Albrecht J., Briem H., Beyer K., Clauβen H., Gastreich M., Lemmen Ch., Rarey M. (2012). J. Comput Aided Mol. Des..

[23] Kuntz I.D., Chen K., Sharp K.A., Kollman P.A. (1999). Proc. Natl. Acad. Sci. USA..

[24] Semenza G. (2012). Cell..

[25] Zheng H., Fridkin M., Youdim M. (2015). Persp. Med. Chem..

[26] Park S.H., Kim B.R., Lee J.H., Park S.T., Lee S.H., Dong S.M., Rho S.B. (2014). Cell Signal..

[27] Schnell P.O., Ignacak M.L., Bauer A.L., Striet J.B., Paulding W.R., Czyzyk-Krzeska M.F. (2003). J. Neurochem..

[28] Hirota K., Ryo Fukuda R., Takabuchi S., Kizaka-Kondoh S., Adachi T., Kazuhiko Fukuda K., Semenza G.L. (2004). J. Biol. Chem..

[29] Epstein A.C., Gleadle J.M., McNeill L.A., Hewitson K.S., O’Rourke J., Mole D.R., Mukherji M., Metzen E., Wilson M.I., Dhanda A. (2001). Cell..

[30] Yuan Y., Hilliard G., Ferguson T., Millhorn D.E. (2003). J. Biol. Chem..

[31] Ma X., Wang X., Cao J., Geng Z., Wang Z. (2014). PLoS One..

[32] Boyko S.S., Zherdev V.P., Dvoryaninov A.A., Gudasheva T.A., Ostrovskaya R.U., Voronina T.A., Rozantsev G.G., Seredenin S.B. (1997). Eksp. Klin. Farmakol..

[33] Gudasheva T.A., Boyko S.S., Akparov V.Kh., Ostrovskaya R.U., Skoldinov S.P., Rozantsev G.G., Voronina T.A., Zherdev V.P., Seredenin S.B. (1996). FEBS Lett..

[34] Fedorova T.N., Us K.S., Ostrovskaya R.U. (2007). J. Neurochemical..

[35] Andreeva N.A., Stemalshuk E.V., Isaev N.K., Ostrovskaya R.U., Gudasheva T.A., Viktorov I.V. (2000). Bull. Exp. Biol. Med..

[36] Zhang Z., Yan J., Chang Y., ShiDu Yan S., Shi H. (2011). Curr. Med. Chem..

[37] Firova F.A. (1994). The range of neurotropic activity of the original substituted prolyl-dipeptide GVS-111. Extended abstract of PhD dissertation (Medicine). Institute of Pharmacology RAMS. Moscow. 1994. (in Russian)..

[38] Ostrovskaya R.U., Vakhitova Y.V., Kuzmina U.Sh., Salimgareeva M., Zainullina L.F., Gudasheva T.A., Vakhitov V.A., Seredenin S.B. (2014). J. Biomed. Sci..

[39] Jia X., Gharibyan A., Öhman A., LiuY. I.O., Olofsson A., Morozova-Roche L.A. (2011). J. Mol. Biol..

[40] Lysenko A.V., Uskova N.I., Ostrovskaya R.U., Gudasheva T.A., Voronina T.A. (1997). Eksp. Klin. Farmakol..

[41] Ostrovskaya R.U., Gruden M.A., Bobkova N.A., Sewell R.D.E., Gudasheva T.A., Samokhin A.N., Seredenin S.B., Noppe W., Sherstnev V.V., Morozova-Roche L.A. (2007). J. Psychopharmacol..

[42] Ostrovskaya R.U., Tsaplina A.P., Vakhitova Yu.V., Salimgareeva M.Kh., Yamidanov R.S. (2010). Eksp. Klin. Farmakol..

[43] Ostrovskaya R.U., Ozerova I.V., Gudasheva T.A., Kapitsa I.G., Ivanova E.A., Voronina T.A., Seredenin S.B. (2013). Bull. Exp. Biol. Med..

[44] Ostrovskaya R.U., Zolotov N.N., Ozerova I.V., Ivanova E.A., Kapitsa I.G., Taraban K.V., Michunskaya A.B., Voronina T.A., Gudasheva T.A., Seredenin S.B. (2014). Bull. Exp. Biol. Med..

[45] Cheng K., Ho K., Stokes R., Scott C., Lau S.M., Hawthorne W.J., O’Connell P.J., Loudovaris T., Kay T.W., Kulkarni R.N. (2010). J. Clin. Invest..

[46] Liu Y., Liu F., Iqbal K., Grundke-Iqbal I., Gong C.X. (2008). FEBS Lett..

[47] van de Velde S., Hogan M.F., Montminy M. (2011). Proc. Natl. Acad. Sci. USA..

